# Designing and validating of a questionnaire measuring perceived self-care ability (PSCA) in chronic stroke patients at home

**DOI:** 10.1186/s12883-024-03612-4

**Published:** 2024-04-15

**Authors:** Nasrin Jafari-golestan, Asghar Dalvandi, Mohammadali Hosseini, Masoud Fallahi-Khoshknab, Abbas Ebadi, Mahdi Rahgozar, Sidani Souraya

**Affiliations:** 1 Faculty of Nursing, Department of Nursing Management, Aja University of Medical Sciences, Tehran, IR Iran; 2 Department of Midwifery, Faculty of Nursing and Midwifery, Islamic Azad University, Tehran, IR Iran; 3 Department of Nursing Education, University of Social Welfare and Rehabilitation Sciences, Tehran, IR Iran; 4 Behavioral Sciences Research Center, Life Style Institute, Baqiyatallah University of Medical Sciences, Tehran, IR Iran; 5 Nursing Faculty, Baqiyatallah University of Medical Sciences, Tehran, IR Iran; 6 Department of Biomedical Statistics, University of Social Welfare and Rehabilitation Sciences, Tehran, IR Iran; 7 School of Nursing, Toronto Metropolitan University, Toronto, Canada

**Keywords:** Perceived Self-Care Ability Questionnaire, Chronic, Mixed method, Psychometric, Stroke

## Abstract

**Background:**

Patients with a stroke often cannot care for themselves after hospital discharge. Assessment of their self-care ability is the first step in planning post-discharge home care. This study aimed to design and validate a measure of perceived self-care ability (PSCA) in stroke patients.

**Methods:**

A sequential-exploratory mixed method was conducted in Tehran, Iran, in 2020–2021. The qualitative phase involved in-depth semi-structured interviews with 12 participants. Transcripts were content analyzed. The results guided the development of 81 items. psychometric properties such as face validity (Impact Score > 1.5), content validity ratio (CVR > 0.63), content validity index (Item Content Validity Index: ICVI > 0.78, Scale Content Validity Index/Average: SCVI/Ave > 0.8) and Kappa value (Kappa > 0.7), internal consistency (Cronbach’s alpha > 0.7), relative reliability (ICC: inter class correlation coefficient), absolute reliability (Standard Error of Measurement: SEM and Minimal Detectable Changes: MDC), convergent validity (Correlation Coefficient between 0.4–0.7), interpretability, responsiveness, feasibility, and ceiling and floor effects were assessed**.**

**Results:**

Content analysis of the qualitative interviews yielded 5 major categories and 9 subcategories that reflected "Perceptual stability", "Cognitive fluctuations", "Sensory, Motor and Physical health",” The subjective nature" and "The dynamic nature” of PSCA. Results of face and content validity reduced the number of items to 32, capturing three dimensions of PSCA in chronic stroke patients; these dimensions included perceptual ability, threatened health status, and sensory, motor, and cognitive ability. The findings supported the reliability and validity of the measure.

**Conclusions:**

The PSCA questionnaire was developed and validated within the Iranian culture. It is useful in assessing the self-care of patients with stroke and in informing practice.

## Introduction

A stroke is a significant and unanticipated event that alters a person's lifestyle, physical performance, and ability to manage self-care, work, and even leisure time [1]. In 2020, strokes caused one death out of every six deaths brought on by cardiovascular problems. In the United States, a stroke occurs every 40 s and results in death every 3.5 min. Strokes lead to long-term disability and impact health and wellbeing. More than half of stroke survivors aged 65 and older experience mobility impairment [2]. Every day, 250 to 300 people in Iran suffer a stroke, which is a higher rate than in Western countries and occurs at a younger age [3]. Early assistance for stroke patients after discharge and continued rehabilitation increases satisfaction with rehabilitation and independence in performing self-care such as mobility, dressing, and personal hygiene, as well lessen the consequences of the illness such as speech impairment and depression [4].

Perceived self-care ability is very important in patients with chronic diseases [5]. It reflects persons’ perceptions of their capacity and aptitude for engaging in self-care [6–8]. Promotion of self-care ability is a perquisite for enhancing people’s engagement in self-care actions and behaviors and overcoming obstacles in self-care [9].

Evidence indicates that the burden of care for stroke patients increases after discharge because of their inability to care for themselves, high dependency on medical services, frequent use of nursing services, and the many people caring for them [5]. And improper planning during discharge and occasionally premature discharge of these patients results in numerous issues for the patients' caregivers [6]. Engagement and continuation of treatment are important issues in the acute and chronic phases of illness [1]. This is because time is crucial in the chain of recovery for stroke patients, which begins from the moment symptoms are identified and continues until rehabilitation. Developing successful therapeutic interventions to support recovery and the effectiveness of rehabilitation programs will be made easier with a thorough understanding of the patient's ability for self-care and measurement of his decision-making power in self-care [7]. Participation and acceptance of responsibility by the patient is a key self-care principle. Given the significant impact of stroke on patients' health, it is essential to assist patients in engaging in self-care behaviors and therefore in promoting functioning and managing complications associated with their illness [8]. It is crucial to use a valid and reliable tool to measure perceived self-care ability in stroke patients because it is subjective concept that cannot be assessed directly [9]. However, there is no tool developed and psychometrically tested to measure this construct in stroke patients [10]. They have to perform self-care behaviors that differ from those expected of patients with other chronic conditions. Tools specifically designed for this purpose will be very helpful in understanding and, ultimately, providing a more thorough assessment of the perceived self-care ability status of stroke patients in charge of their own self-care activities at home. For nurses, health policy makers, and family caregivers of this group of patients, accurate measurement of this ability will open up the possibility of effective care planning and interventions to enhance quality of life. Based on this, the researcher conducted a study to design and validating a questionnaire to measure the perceived self-care ability of stroke patients at home to fill the knowledge gap that existed.

## Materials and methods

A sequential mixed-method design was used. It involved a qualitative phase to explore the dimensions of self-care as reported by patients with stroke. The results formed the basis for developing items measuring perceived self-care ability. The quantitative phase aimed to examine the reliability and the validity of the measure in a sample of patients with stroke residing in Tehran, Iran, in 2020–2021.

### Qualitative phase

Since the perceived self-care ability in stroke patients is a context-based construct, [11] as noted in the introduction section, stroke patients have special conditions compared to other chronic patients. Their debilitating nature of stroke can affect all aspects of a person's life with a chronic stroke disease. These special conditions, including dependence on others, lead to changes in the perceived self-care abilities. Thus, in the first phase of the study, a qualitative method was used to understand the construct of the perceived self-care ability and its dimensions in stroke patients living at home and receiving rehabilitation. Qualitative methods were used to collect and analyze data. The methods are appropriate in the stage of developing measures [12]. In-depth and semi-structured interviews were conducted with 12 participants selected by purposive sampling. Purposive sampling was used to represent patients with different socio-demographic and health characteristics such as (age, gender, education, job, marital status, and frequency of stroke attacks). Sampling was stopped when information saturation was reached. 12 eligible patients provided informed consent, and the concurrent analysis of their responses reached saturation. Most patients were interviewed in a private, quiet location at the rehabilitation center, at their convenience. Key participants of this study were those over 18 in chronic phase of stroke (were within the one-month period following discharge from acute care hospitals), had the most involvement in self-care, had rich experiences and could talk about the research question. The exclusion criterion was refusal to continue participation. The participants were informed about the details of the study, and confidentiality of any disclosed information. Patients were assured that withdrawal from the study did not affect providing care. Written informed consent was obtained from all the participants. At the beginning of each interview, they were assured of the confidentiality of their information**.** The transcripts of the interviews were content analyzed using the conventional approach, following the five-step method by Granheim and Lundman (2004) [13].

### Quantitative phase

In the second phase of the study, a cross-sectional study was used to examine the psychometric properties of the perceived self-care measure.

### Face and content validity

To determine the content validity, 15 stroke patients and 12 experts were asked to rate the relevance of the items’ content in capturing self-care and to comment on the questionnaire. Participants were selected using convenience sampling. The data were analyzed using different validity indices (with established cut-off values), including: regarding face validity (Impact Score > 1.5), content validity ratio based on Ayre and Scally’s12 table (CVR > 0.63), content validity index (Item Content Validity Index: ICVI > 0.78 & Scale Content Validity Index/ Average: SCVI/Ave > 0.8), and Kappa value (Kappa > 0.7). Finally, the final decisions were made by the indices mentioned above and comments collected from the research team on the deletion, modification, and inclusion of the items. To calculate the Scale Content Validity Index (SCVI), we first calculated the ICVI value for each item in the inventory, and then the mean of total ICVI was calculated for all items [14]. The results guided the decisions to delete, modify or include / keep items in the measure.

### Item analysis

Before Factor Analysis, a preliminary study was conducted on 50 of the key participants to assess the adequacy of the items and to spot distorted items using the Discrimination Index and Loop Method. Its assumptions must be verified to perform construct validity [14]. To accomplish this, the samples' adequacy was evaluated using the Kaser-Meyer-Olkin (KMO) formula [15, 16], and the correlation matrix between the items was examined using Bartlett's Test of Sphericity. The correlation between each item's score and the test's total score was discovered to check the discrimination index. The item was eliminated if the correlation coefficient between it and the entire questionnaire was less than 0.3. And one item was eliminated if the correlation coefficient between two items was higher than 0.7. The reliability coefficient of each question was calculated before applying the Loop Method or Inter Item Total Correlation. This question is appropriate because it effectively coordinates with other questions, as shown by the fact that the level of reliability decreased when questions were removed [14].

### Construct validity

The Factor Analysis Method is among the best methods for measuring construct validity [14]. According to the rule of thumb of having 5–10 cases per item, 280 stroke patients who met the study's inclusion criteria (including being over 18 and having had a stroke for at least two weeks) [9], were chosen by convenience sampling [13]. Latent factors were then extracted using exploratory factor analysis, principal axis factoring, and Varimax rotation. The SPSS software, version 24, was used to perform the statistical analysis.

### Convergent validity

The instrument used in this validity must converge with other instruments used to measure the same construct. This can be achieved if the Pearson correlation coefficient is higher than 0.4 [13]. The 17-item SASE questionnaire (The Self-care Ability Scale for the Elderly) was also filled out by the 50 participants who were chosen by random sampling method in the study to measure self-care ability in stroke patients at home. Soderhamn in Sweden validated and approved this questionnaire in 2001. This questionnaire is based on Porn's (1993) theory of health and adaptedness, which has three key components: repertoire, environment, and goal. This self-report questionnaire assesses the elderly's perceived self-care ability. It demonstrated good reliability (Cronbach's α = 0.68), face validity, predictive validity, concurrent validity and construct validity in elderly patients [17]. However, Soderham's study (1996) showed that the self-care ability questionnaire from the perspective of the elderly did not have the necessary sensitivity for the elderly with diseases such as stroke, and for people with chronic and underlying diseases, redefining the concept of self-perceived care ability has been necessary and this concept needs a clear and operational definition and on the other hand, the existing tools that are designed in the field of old age are in the context of Scandinavian countries and there is a need to create specific tools with the cultural background of other societies, due to the nature of dependent on The environment and culture of the concept of perceived self-care ability are necessary (22). Since it is not advised [18]. for there to be very strong correlations between the results of the two tests, the correlation between the present questionnaire and the elderly self-care ability questionnaire, which consists of 17 questions, was deemed favorable (P = 0.543).

### Reliability

The questionnaire's reliability was assessed using the internal consistency and stability consistency methods. Absolute and relative stability are both parts of stability. Inter Class Correlation (ICC) was calculated to assess relative stability, and Standard Error of Measurement (SEM) and Minimal Detectable Changes (MDC) were calculated to assess consistency in stability.

### Internal consistency

Correlation between the items of an instrument is called internal consistency. To assess internal consistency or homogeneity, Cronbach's alpha was calculated. For a question to be kept in an instrument, the alpha value must be at least 0.7 [18].

### Stability consistency

In this study, the tool was administered twice to the same samples, two weeks apart, and then intra-cluster correlation analysis was performed between the scores of the two tests. Also, the correlation coefficient of 0.8–0.9, which indicates good reliability was considered [19].

### Standard error of measurement

The standard deviation in this study was determined using the equation shown below:

$${\text{SEM}}={\text{SD}}\sqrt{1-{\text{ICC}}}$$

The coefficient of repeatability (ICC) and standard deviation (SD) are both terms used in the Eq. [14].

### Minimal detectable

The following equation was used to determine the Minimal Detectable value:

$$\mathrm{MDC }=\mathrm{ SEM }\times {\text{z}}\times \sqrt{2}$$

To determine the relative true changes following treatment or between repeated measurements over time, and to demonstrate the relative amount of random measurement error, the minimal detectable change can be calculated as a "percentage of minimal detectable change" as shown in the following equation.

$$\mathrm{MDC\%}=\left({\text{MDC}}\div {\text{mean}}\right)\times 100$$

Less than 30% of the "percentage of minimal detectable change" is considered acceptable, and less than 10% is thought to be excellent [14, 18].

### Responsiveness

In terms of responsiveness, we anticipate that the target structure can demonstrate how people's conditions change over time, either for the better or worse [20]. Hypothesis testing is one method for figuring out responsiveness [14] The method mentioned above was applied in this study to assess the instrument's responsiveness. The score change in the two groups was determined after the tool was administered to two groups of stroke patients (280 people) with varying conditions (based on the time that had passed since the stroke).

### Interpretability

In the score of the tool, "Interpretability" is defined as the qualitative significance of "Minimal Important Changes" [14]. The criteria for interpretability based on the COSMIN checklist include calculating the minimal important change, figuring out the ceiling and floor effect, interpreting the distribution of total scores in the samples, figuring out the percentage of Missing Item, and determining the adequacy of the sample size [21].

### Minimal important changes

In this study, the standard deviation of the changes between Test–Retest was multiplied by the average effect size, which is 0.5 [20, 21], and MIC should be greater than MDC [14] to calculate the minimal important change.

### Ceiling effect and floor effect

The ceiling effect happens when most respondents select the options at the upper limit of the scale, and the floor effect happens when most respondents select the options at the lower limit of the scale. The researcher can rewrite these items in "strongly positive" and "strongly negative" to make them harder to improve them [17]. To consider all factors and demonstrate changes over time, this index must be less than 20% [22]. The ceiling effect and floor effect were calculated for the total score of the questionnaire and the score of all subscales in this study to assess the discrimination power of the questionnaire and distributing the answers.

Examining the distribution of scores in the samples is another way to confirm interpretability. For instance, different groups will have different response variable mean and standard deviation values. Based on this, the questionnaire's designed questions were used to calculate the average perceived self-care ability of the participants in the current study across various classes.

### Feasibility

The average time to complete the questionnaire and the percentage of people who did not answer every question are two criteria measured to determine the feasibility. The questionnaire should be simple and simple to complete. Most questions should have an unanswered rate of less than 1%. Performance measurement defines the instrument's feasibility, usability, and applicability. Age, language, culture, cognitive state, and ability of samples or patients must all be considered for a tool feasible.

### Weight assignment

Ranking of items is one of the most practical applications of factor analysis.

$$\mathrm{Transformes}\;\mathrm{score}=\frac{\mathrm{Actual}\;\mathrm{raw}\;\mathrm{score}-\mathrm{Lowest}\;\mathrm{possible}\;\mathrm{raw}\;\mathrm{score}}{\mathrm{Highest}\;\mathrm{possible}\;\mathrm{raw}\;\mathrm{score}-\mathrm{Lowest}\;\mathrm{possible}\;\mathrm{raw}\;\mathrm{score}}\times100$$

There are essentially two approaches for weighting and ranking items. The first approach involves using the field survey method, which involves surveying professionals and knowledgeable individuals. The second approach involves using statistical methods, which are based on selecting a group of items, looking at their statistical structure, figuring out how correlated they are, and then assigning weights that fit the data as it was observed. By having the loading of each factor and the weight of each item in each factor, the importance and weight of each item can be determined through the relation given below. In this step, the factor loading of each item was the weight and importance of each item in that factor.

$${WI}_{ij}={W}_{fi}\times {P}_{fiIj}$$

$${w}_{fi}=\frac{{\lambda }_{i}}{\sum_{j=1}^{k}{\lambda }_{i}}\times {100}$$

Some items in the factor analysis may not be present in the factors chosen. This is because that item only makes up a small portion of the variance in the entire scale [23].

### Scoring items

Options on this questionnaire have a Likert scale with a maximum of five points (always 5, often 4, sometimes 3, rarely 2, never 1). This tool has a scoring range of zero to one hundred. The scores obtained using the linear scoring method are transformed into standard scores for this purpose using the formula below. The person's perceived self-care ability increases with the score they receive using the current tool.

Thirty two items were finally designed for a perceived self-care ability tool for stroke patients at home after two qualitative and quantitative stages (psychometrics).

## Results

### Qualitative phase

Four hundred eighty one primary codes, 26 subcategories, and 11 main categories were found in this study's qualitative phase. The codes were combined, and five main categories and nine subcategories with the headings "Perceptual stability", "Cognitive fluctuations", "Sensory, Motor and Physical health", "The subjective nature of the phenomenon of perceived self-care ability" and "The dynamic nature of the phenomenon of perceived self-care ability" were produced (Table 1).

**Table 1:** The initial codes, subcategory and main category of attributions the perceived self-care ability concept in stroke patients (Analysis of texts)

Initial codes	Main category	Sub category
Ability to perception	Perception ability	Perceptual stability
Ability to prioritize needs		
Ability to perceived changes in life conditions		
Visual perception		
Individual attitude towards health	The perceived to threat	
Understanding the threat ahead		
Self-perception		
Understanding yourself as a self-care agent		
Unmet expectations of the itself		
Awareness of time, place and person	Mental and cognitive ability	Cognitive fluctuations
Recognition of individual abilities		
Seek appropriate help to meet needs		
Awareness of the conditions that arise		
Ability to sense	Sensory ability	Sensory, Motor and Physical health
Ability to touch		
Health in the five senses		
Strength of movement of upper and lower limbs	Motor ability	
Ability to walk		
Ability to perform daily life activities		
Physical strength	physical ability	
Not having multimorbiditi		
Ability to maintain balance while sitting and standing		
Structural health of the body (body anatomy, structure of the brain and organs		
Processing information related to the disease•Perceived disability	Perceived of problem	The subjective nature of the phenomenon of perceived self-care ability
Integration of the current experience of the disease with previous experiences		
Acceptance of disability		
Treatment adherence	The ability to improve health	The dynamic nature of the phenomenon of perceived self-care ability
Participation in self-care activities		
Efforts to maintain health		
Trying to learn self-care skills		
Choosing a healthy lifestyle		
Promote recovery over time	Evolutionary process and move forward	
Fulfilled expectations of yourself		
The desire to regain the self-care ability		

The capacities and capabilities that a person with a disability has to understand how to take care of themselves are included in these five main categories, which have nine subcategories.

These five main categories have been repeatedly mentioned in the texts, both directly and indirectly. The perceived self-care ability in patients is therefore influenced by their level of mental, cognitive, perceptual, and physical or functional development.

### Quantitative phase

#### Determining validity

The number of items was reduced from 81 to 47 items at the conclusion of this stage, based on calculations made during the face and content validity stage and the opinions of the evaluators and the research team. The entire instrument's average content validity index in the current study was 0.89. Typically, instrument makers accept an index score of 0.9 as an excellent criterion and an index value of 0.8 as the lower limit of acceptance for the instrument's content validity [14].

### Item analysis

Cronbach's alpha was 0.888 at this point. In all cases, the correlation coefficient between the two items was under 0.7. There were only 41 items left instead of the original 47. The following is a list of the reduced items:

1. I have trouble taking care of myself.
2. Other people's attention and follow-up have an impact on my abilities.
3. I'm aware of time, place, and people in my surroundings.
4. I feel things by touching them.
5. Without assistance from others, I am unable to care for myself.
6. Without assistance, I am unable to maintain normal blood pressure and blood sugar levels.

### Factor analysis

Principal-component analysis and convergent validity were used in this study to assess the construct validity of the perceived self-care ability structure, which consisted of 41 items. In this study, factorization was carried out using Orthogonal Varimax rotation and Principal Components Analysis to create clusters. Kaser-Meyer-Olkin (KMO) was calculated to assess the adequacy of the samples, and Bartlett's Test of Sphericity was applied to assess the correlation matrix between the items.

The significance of Bartlett's sphericity test also revealed there is sufficient correlation between the items [14]. Table 2 demonstrated that the KMO index is higher than 0.5, indicating that the data are sufficient. The test's latent factors were then extracted using a principal-components factoring method based on Orthogonal Varimax rotation.

**Table 2:** KMO sampling adequacy index and Bartlett test results

**Kaiser–Meyer–Olkin Measure of Sampling Adequacy**		**0.884**
Bartlett’s Test of Sphericity	Approx. Chi-Square	5592.071
df	428	
Sig	*P* < 0.001	

**Fig. 1:**
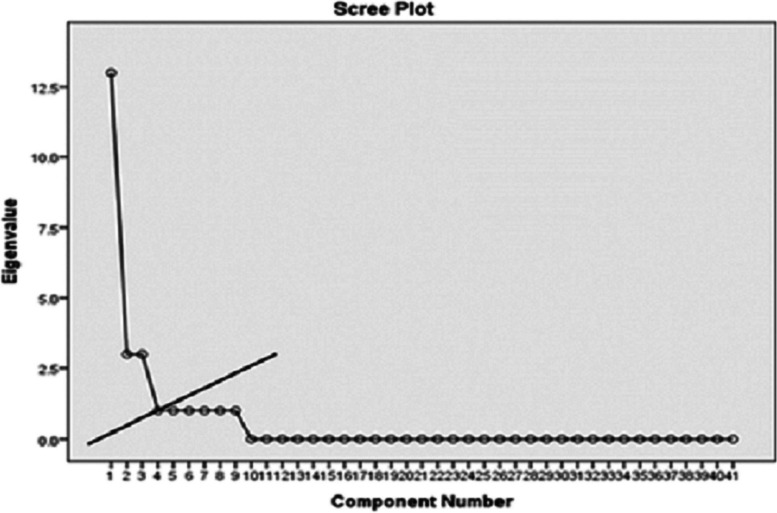
Determining the number of factors constructing the questionnaire measuring the perceived self-care ability of stroke patients at home

According to the Scree plot diagram and the Eigenvalue greater than 1, five factors from this model were extracted (Fig. 1). The 3-factor model, however, had a better fit in terms of the logic underlying item arrangement and labeling after performing factor analysis 10 times. The three latent factors were 7.48, 4.68, and 4.62, respectively. The three extracted factors (Table 3) accounted for 52% of the variance in the variables measured by the self-care ability questionnaire in stroke patients.

**Table 3:** Labeling each factor based on the relevant items by factor loading

No	Items	Factor loading		
		Factor I	Factor II	Factor III
1	I need help from others to get in and out of bed	0.823		
2	I need help from others to ascend and descend the stairs	0.815		
3	I take my medications on my own, as directed by the doctor	0.805		
4	I need help from others to roll over in bed	0.789		
5	I am able to provide a detailed report of the progress of my health condition to the doctor	0.749		
6	I am able to provide a detailed report of the progress of my health condition to the doctor	0.736		
7	I am still able to provide a comfortable lifestyle for myself despite my disability	0.728		
8	I am aware of the signs and symptoms of a possible recurrence of the disease	0.691		
9	My past experiences keep me in good health	0.655		
10	I have a say in self-care decisions	0.597		
11	Despite my disability, my self-care skills are enhancing day by day	0.561		
12	I can perform daily tasks outside the house (shopping, attending family parties)	0.540		
13	I can do daily life activities (bathing, going to the toilet, preparing a simple snack, dressing and doing personal hygiene)	0.498		
14	I control my mental pressure and stress	0.496		
15	With the help of something or someone, I can take ten steps around the house	0.462		
16	I know who to ask for help in case of a problem	0.445		
17	Given the current situation, I need more help from my family and society		0.888	
18	Families endure a lot of hardship to take care of me		0.840	
19	I am worried that the disability might be permanant		0.758	
20	I need more time to fully understand my disability		0.729	
21	I worry about relying on the help of others		0.687	
22	I feel that the family pays attention to my opinions		0.644	
23	Spiritual beliefs (prayer, etc.) help me deal with my disability		0.556	
24	I rely on the abilities I still have		0.493	
25	Both when sitting and standing, I can maintain my balance			0.838
26	I can be drawn in by the conversations going on around me			0.824
27	I am able to see and identify objects around me			0.804
28	I am in control of my bladder (urine and feces)			0.804
29	I am able to pronounce the words in order and comprehend their meaning			0.772
30	I can move my arms and legs			0.626
31	My ability to move is limited by numbness in my hands and feet			0.565
32	I am afraid of the future			0.496

Factor I: sensory, motor and cognitive ability, Factor II: cognitive ability, Factor III: Threatened health status

The above table shows that some items with various factor loadings were cross-loaded into several factors. The majority of these were placed in the factor with the highest factor loading to maximize factor loading; however, some items were removed because they did not match the factor with the highest factor loading or common factor loading. Nine items, including items 4, 8, 11, 13, 14, 22, 15, 31, and 32, were eliminated.

The statement "I feel that I am ready to take care of myself at home" was deleted. And the statement "Now when I have a problem, I find a solution to it" was taken out. And the statements "I am aware of the important skills for self-care" and "I work to acquire the knowledge and abilities required for self-care", "The important goal of my life is to be independent", "I use all my power to take care of myself", "I feel alienated from the injured side", "I cooperate in doing rehabilitation activities (occupational therapy, speech therapy, physiotherapy, etc.)" and "I understand the disability that happened to me" were taken out of the 41-question questionnaire. 32 items with three latent factors were formed using the findings of the factor analysis. Ability in terms of sensory, motor, and cognitive ability came first. From factor 2 to factor 1, item 12 was moved. The factor number two, perceptual ability, had eight items. The threatened health status was the factor three.

### Determining reliability

#### Internal consistency

The Cronbach's alpha values for each factor and the entire questionnaire were estimated to be favorable, as seen in the table below (Table 4).

**Table 4:** Determination of Cronbach's alpha coefficient by the dimensions and the whole questionnaire

**Factor**	Label	No. of items	Cronbach’s alpha Coefficient
Sensory, motor and cognitive ability	1	16	0.948
Perceptual ability	2	8	0.734
Threatened health status	3	8	0.743
Total		32	0.901

#### Stability consistency

Table 5 shows that for each factor and the entire instrument, the Inter Class Correlation value is close to one and the lower limit of the confidence interval is high. Therefore, the current tool has good reliability.

**Table 5:** The absolute reliability of the perceived self-care ability tool in stroke patients at home

**Factor**	**mean**	**SD**^a^	**ICC**^b^	**CI**^c^** = 95%**	**SEM**^d^	**MDC**^e^	**MDC%**	**Result**
Sensory, motor, cognitive ability	50.66	12.1	0.94	0.874 – 0.975	2.76	15.04	7.62	Acceptable
Perceptual ability	32.27	5.40	0.74	0.416 – 0.884	2.74	23.45	7.57	Acceptable
Threatened health status	32.33	3.86	0.73	0.419 – 0.879	1.98	16.91	5.47	Acceptable
Total	115.27	19.43	0.849	0.762 – 0.953	5.26	12.60	14.53	Acceptable

^a^Standard Deviation

^b^Interclass Correlation Coefficient

^c^Confidence Interval

^d^Standard Error of Measurement

^e^Minimal Detectable Changes

### Absolute reliability

#### Convergent validity

Correlation The 17-item self-care ability questionnaire for the elderly had a coefficient of 0.543 between it and perceived self-care ability in stroke patients at home, indicating good convergent validity.

### Responsiveness

The ANOVA test results revealed there is a significant difference in the perceived self-care ability between the three groups of patients (those who have had a stroke for a certain amount of time, such as two weeks to one month after the stroke, one month to one year later, and one year more), depending on when the stroke occurred. The post-hoc LSD test also revealed that patients with the disease for a longer while perceived their ability to care for themselves as being significantly better than patients with the disease in its early stages.

One year and above duration of stroke (F = 11.54, df = 2, *P* < 0.001).

### Interpretability

#### Determining the ceiling and floor effect

Table 6 show the Determining the ceiling and floor effect.

**Table 6:** Determining the effect of ceiling and floor for the whole and separately for each dimension of the questionnaire

Ceiling effect (percentage)	Frequency of maximum score	Floor effect (percentage)	Frequency of minimum score	Factor
0.5	1 person	0.5	1 person	Total tool
0.5	1 person	0.5	1 Person	Sensory, motor, cognitive ability
6.3	13 persons	2.4	5 Person	Perceptual ability
0.5	1person	1.5	3 Person	Threatened health status

### Examining the distribution of scores

Examining the distribution of scores in the samples is another way to confirm interpretability. For instance, different groups will have different response variable mean and standard deviation values. Based on this, the questionnaire's estimated average self-care ability for participants in the current study was divided into various classes.

### Weighting of items

The weight of each item in the perceived self-care ability questionnaire for chronic stroke patients receiving care at home was first calculated to determine the actual weight of each item and rank the questions in each dimension. The factor loading of each item was multiplied by the proportion of each factor's variance to the total variance, which came to 52.481. The weight of each item was then determined by dividing the secondary values at this stage by the sum of secondary values (862.88).

As seen, the sensory, motor, and cognitive ability dimensions contain the items with the highest weights. And the items that explain a particular dimension the best are those that carry the most weight in that dimension.

The difference between the raw and weighted scores was calculated using the non-parametric Wilcoxon test after the item weights were determined, the Kolmogorov–Smirnov normal distribution test was run, and the significance of P was determined (the null hypothesis that the distribution is normal is rejected). The test was done in raw and weighted form. Since the results indicate a significant difference between the two cases, each item's score must be multiplied by its own weight to determine the final score (Table 7).

**Table 7:** Comparison of the average standard scores obtained from the questionnaire in two modes of raw and weighted

**Average scores in two modes of raw and weighted**	**SD**	*P* value
111.16	20.30	0.001

### Feasibility

#### Scoring

Options on the questionnaire have a 5-point Likert scale (always, often, sometimes, rarely, and never). This tool offers scores between 32 and 160. The person's perceived level of self-care ability increases with the score they receive using the current tool. The score for the option is never (1), often (2), sometimes (3), rarely (4), and always (5). Items 27 through 29 are scored in the reverse order. A weighted questionnaire should be designed and used.

### Linear transformation

The scores from the questionnaire were determined and verified using a linear transformation of the score. The scores obtained using the linear scoring method are then converted to a standard score between 0 and 100 for this purpose (26). The person's perceived level of self-care ability increases with the score they receive using the current tool.

$$\text{Converted score}=\frac{\text{Real raw score}-\text{The lowest possible raw score}}{\text{The highest possible raw score }- \text{The lowest possible raw score}} \times {100}$$

## Discussion

The current study was to develop and psychometrically assess a self-care ability questionnaire for stroke patients at home. The findings from the qualitative stage demonstrated that chronic stroke patients' perceptions of self-care at home are characterized by five factors: perceptual stability, cognitive fluctuations, sensory, motor, and physical health; the phenomenon of perceived self-care is subjective; and the phenomenon of perceived self-care is dynamic.

The first characteristic of the concept of perceived self-care ability in chronic stroke patients at home is perceptual stability. The necessity of two hidden and main presuppositions, namely perceptual ability and cognitive ability, has received a lot of attention in the theoretical stage. Despite being in the survey phase, these two presuppositions were not extracted independently. Due to their broadness and inclusion of numerous characteristics, perceptual and cognitive ability serve as an umbrella concept that covers other concepts discovered during this study. One factor that predicts one's ability for self-care is perceptual ability. Combining perception and motor activity can improve one's self-care ability [7]. Information is received through the senses and translated based on one's worldview during the dynamic process of perception. To dress, for instance, one must be able to comprehend and correctly identify the body part [24]. In the first five weeks following an attack, cognitive anilities strongly correlate with self-care in stroke patients [25], which followed the findings of the current study. Patients in the current study held the view they could better deal with and adapt to disabilities if they had a greater understanding of problems and disabilities. Meanwhile, self-belief, relying on residual abilities following illness, and learning motivational approaches will all be important factors in successfully accepting disability.

Cognitive fluctuations are defined as fluctuations in thinking, learning, problem-solving, and memory abilities; they can affect one's physical, mental, social, and spiritual abilities [26]. Disturbances in cognition can cause problems with concentration, recall, learning, planning, manipulating information, beginning and ending activities, using language, and recognizing mistakes [24].

To engage in conscious activities and self-care behaviors, one needs to be in good physical, sensory, and motor health and to have good perceptual and cognitive abilities [27]. The ability to walk, move, stand, and use one's hands were frequently used as synonyms for the self-care ability by patients in the current study.

Another aspect that patients frequently brought up during the survey phase was the subjective nature of the phenomenon of perceived self-care ability, or the inherent understanding of the problem. They took cared of themselves less effectively after the disease because of its chronic and debilitating nature, and they clearly understood this. According to some researchers, the perceived issue is actually the perceived disability, which is subjective [28].

According to the findings of other studies, the more a person knows his self-care limitations, disabilities, and reliance on others, the better aware he is of his ability to care for himself [29] and the more freely he can choose a self-care behavior that will meet his needs [30], and recognize and apply other compatible and incompatible influencing factors to match his disabilities at this stage [31].

The perceived self-care ability is correlated with normal perception, physical health, and sensory functions [25].

Although some researchers contend that it is preferable to focus on patients' abilities rather than overly relying and emphasizing people's disabilities, this issue is more valuable and important in hastening the recovery process of stroke patients [29] and feeling helpless can cause a sense of helplessness and a sense that one cannot control the environment, which can cause frustration and subsequent aggressive behaviors [32]. Two subjective and dynamic aspects of the concept of self-care ability implicitly understood in two characteristics, the perceived problem and the perception of threatened health, were extracted during the analysis of the survey stage results. Patients could not directly refer to the concepts of subjectiveness or dynamics of the concept. However, they clarified it in their statements they deeply felt the inability to take care of themselves. They also acknowledged that their circumstances had changed and that they needed to pass the time for their condition to improve with time. The core of social participation for these patients is their capacity to comprehend and accept stroke-related issues, adapt to them in terms of behavior and attitude, and make the right choice based on self-management skills [33].

According to the results of the current study, people's perceptions of their ability to take care of themselves can vary depending on their values, beliefs, feelings, and types of thoughts, and depending on each disease and its effects [34].

Following psychometric procedures, a self-care questionnaire with 32 items and 3 dimensions of sensory, motor, cognitive, perceptual ability, and threatened health status was given to stroke patients at home. Sensory, motor, and cognitive ability is the first factor, and it consists of 16 questions about "functional abilities, mobility, performing daily life activities, compliance with treatment, control of psychological pressures, and adaptation to the disability needed by the individual to understand the level of ability to take care of oneself." Self-care abilities are viewed as requiring sensory, motor, and cognitive abilities [35, 36].

The "Sensory, Motor, Cognitive Ability" factor correlates with the overall perceived self-care ability score among the three factors taken from the perceived self-care ability questionnaire in stroke patients, while the "Threatened Health Status" factor has the lowest correlation. The results of the studies also demonstrated that getting into bed and out of bed with one hand or without assistance from others is one of the most valuable functional abilities for self-care in stroke patients [37]. The factor of sensory, motor, and cognitive ability, compared to other factors, can therefore be concluded to be more important in explaining the concept of perceived self-care ability in stroke patients. With the most items and weights assigned, sensory, motor, and cognitive ability is one of the key dimensions of this tool. Physical dependences, such as moving around in bed and performing such activities, are also one of the main causes of long-term stays for stroke patients in care facilities and their hospitals because these activities cause weakness and fatigue in these patients, result in their double disability, and have a significant impact on their quality of life [25]. According to studies, there is a strong correlation between physical performance limitations and the time patients spend in hospitals, and this correlation is more pronounced in the self-care dimension [38].

This tool's second factor consists of 8 questions that measure "ability to perceive disability, acceptance of disability over time, need for social support, burden feeling on family, relying on spiritual beliefs in coping with disability, and ability to solve problems to better understand one's ability to take care of oneself." "Perceptual ability" is the name of this factor. Comparing the correlation between the questionnaire's overall score and the dimension of cognitive ability to the dimension of perceived health status, there wasn't much of a difference. The weight of the items making up this dimension also ranged from at least one to a maximum of two. According to the results of the studies, one of the most crucial factors affecting one's self-care ability is the ability to maintain balance while sitting and standing [25]. Patients have stated in some studies that having poor balance is one of the main causes of their reliance on other people's assistance or using mobility aids like canes and walkers. The fear of falling while walking because of poor balance lowers self-confidence, which increases dependence in patients [39, 40]. Typically, 65% of patients have difficulty using their hands and upper limbs normally six months after a stroke, which affects their ability to balance [41].

Threatened health status is the third dimension or factor. 8 questions make up this factor, which is about "understanding the change in health conditions, trying to promote health, ability to prioritize needs, understanding limitations, and relying on the individual's remaining abilities to better understand the ability for selfcare." With the correlation between this factor and the questionnaire's overall score, it was not much different from the second factor, or perceptual ability.

According to the weights assigned to the questionnaire items, it was discovered that they ranged from 1 to 6. For patients, items with a higher weight are more crucial, while those with a lower weight are only second in importance.

An important finding of this study is that, although the entire questionnaire has a favorable relative reliability, the lower limit for the dimensions or classes related to factors number 2 (perceptual ability) and number 3 (perceived health status), for which the lower limit should be at least 0.6, is approximately 0.4, indicating there is a problem in this part. According to the researcher's stage-by-stage experience, one cause for the low intraclass correlation of these two factors or areas may be the existence of hidden cognitive and perceptual fluctuations during the illness of stroke patients. Because of this finding, the researcher decided to repeat the measurement of this stability by completing 20 questionnaires spaced two weeks apart.

The reanalysis's findings also showed that the questionnaire's relative stability in the two areas of perceived health status and cognitive ability, such as the area of sensory, motor, and cognitive ability, was unfavorable. Compared to the first area, these two may have a greater impact on a person's level of perceptual and cognitive ability.

One of the study's limitations was that it was impossible for the researcher to visit the patients' homes, making it impossible to observe how their lives were going. Perceptual and cognitive fluctuations occasionally made the interview process difficult, and the interviewer had to carefully and patiently identify these circumstances before deciding whether to keep going with the interview or let the patient finish the questionnaire. Confirmatory factor analysis should also be used to strengthen the findings of this study.

Every self-reporting tool has its weaknesses and limitations, including the questionnaire used in this study. The researcher could not control and modify the hidden and complex problems in perception and cognition that stroke patients experience.

## Conclusion

The present study's findings demonstrated that perceived self-care ability in stroke patients has a variety of dimensions to consider when providing nursing care, developing interventions, and developing rehabilitation programs. The results of the present study allow us to conclude that patients with chronic and disabling diseases like stroke should have their perceived level of self-care ability given more weight. Few studies have specifically addressed this concept in stroke patients in a qualitative and in-depth study. The data from this study has given a clearer and complete picture of this concept and can serve for further study in this field. A questionnaire gauged perceived self-care ability in stroke patients based on patient experiences with the concept and its various dimensions. Based on the findings of this study and the advice of nursing professionals with expertise in the care of stroke patients, effective measures can be taken to identify self-care needs, assess the level of self-care disabilities, and remove barriers these patients face when they are responsible for their own self-care at home.

## Data Availability

The data and materials associated with the manuscript will be available upon reasonable request.

## Abbreviations

PSCA: Perceived self-care ability
CVR: Content Validity Ratio
CVI: Content Validity Index
SCVR: Scale Content Validity Ratio/Average
SCVI: Scale Content Validity Index
ICC: Interclass Correlation Coefficient
SEM: Standard Error of Meagerment
MDC: Minimal Detectable Changes
ICVI: Item Content Validity Index
KMO: Kaser-Meyer-Olkin
SASE: The Self-Care Ability Scale for the Elderly
SD: Standard Deviation
MIC: Minimal Important Changes
COSMIN: Checklist Include Calculating the Minimal Important Change
ANOVA: Analysis of variance
LSD: Least Significant Difference Test
